# Cutaneous Leishmaniasis Requiring Polymerase Chain Reaction (PCR)-Based Diagnosis

**DOI:** 10.7759/cureus.98161

**Published:** 2025-11-30

**Authors:** Guadalupe Mercado, Charlotte Read, Cory L Simpson

**Affiliations:** 1 Osteopathic Medicine, College of Osteopathic Medicine, Touro University Nevada, Las Vegas, USA; 2 Department of Dermatology, University of Washington, Seattle, USA

**Keywords:** cutaneous leishmaniasis, diagnostic molecular pathology, leishmania braziliensis, protozoan infections, tropical medicine

## Abstract

Cutaneous leishmaniasis, a protozoal skin infection transmitted by the bite of infected female phlebotomine sandflies, can present a diagnostic challenge outside endemic regions. We describe a twenty-nine-year-old woman who recently emigrated from Ecuador and presented with an enlarging ulcer on the left thigh that failed to respond to multiple courses of antibiotics. Histopathology and tissue culture from skin biopsies were nondiagnostic, but polymerase chain reaction (PCR) testing identified *Leishmania braziliensis*. Intravenous liposomal amphotericin B resulted in complete ulcer resolution within two months. This case highlights the importance of considering leishmaniasis as a diagnosis for non-healing skin ulcers in patients from endemic areas and underscores the value of molecular pathogen identification.

## Introduction

Leishmaniasis is a protozoal disease that can present with a wide range of clinical manifestations depending on the species of Leishmania and the host immune response. The disease has classically been divided into Old World and New World forms based on the geographic distribution of its vectors [1], which is expanding with changes in climate. Though both forms cause cutaneous lesions, New World species carry a higher risk of mucosal disease, while Old World species more often progress to visceral leishmaniasis [2-4]. The cutaneous lesion typically begins as a skin papule or pustule at the site of inoculation by a sandfly bite that ulcerates [5]. Depending on the infecting species, cutaneous ulcers may self-resolve within three to 18 months; however, about 10% become chronic and disseminate, particularly in immunocompromised patients [5-8]. Species identification is critical to prognosis and treatment decisions. For example, *Leishmania braziliensis* infections have a very low spontaneous resolution rate and a higher risk of mucocutaneous progression, warranting systemic therapy, while *Leishmania mexicana* commonly self-resolves. Between 700,000 and 1.2 million new diagnoses are made annually, though cases are likely underestimated due to missed diagnoses and underreporting [9,10]. The diagnosis of cutaneous leishmaniasis is often delayed in non-endemic settings due to low clinical suspicion despite the large global burden of disease. Importantly, increased human migration and displacement necessitate recognition of this disease by clinicians who have trained and who practice outside of traditional endemic regions [9,10].

## Case presentation

A 29-year-old woman who recently emigrated from Ecuador presented to the Emergency Department (ED) with an enlarging, painful ulcer on her left thigh that began as a pustule three months prior. The patient reported no significant medical history and denied any preceding trauma, known insect bite, or use of topical therapy. In the ED, she was prescribed 10 days of trimethoprim-sulfamethoxazole (800 mg-160 mg twice daily) plus cephalexin (500 mg twice daily) for suspected cellulitis. The ulcer continued to enlarge, prompting a second ED visit two weeks later. An ultrasound ruled out an underlying abscess. The patient was prescribed seven days of doxycycline (100 mg twice daily) and was referred to the Dermatology clinic two weeks later.

**Figure 1:**
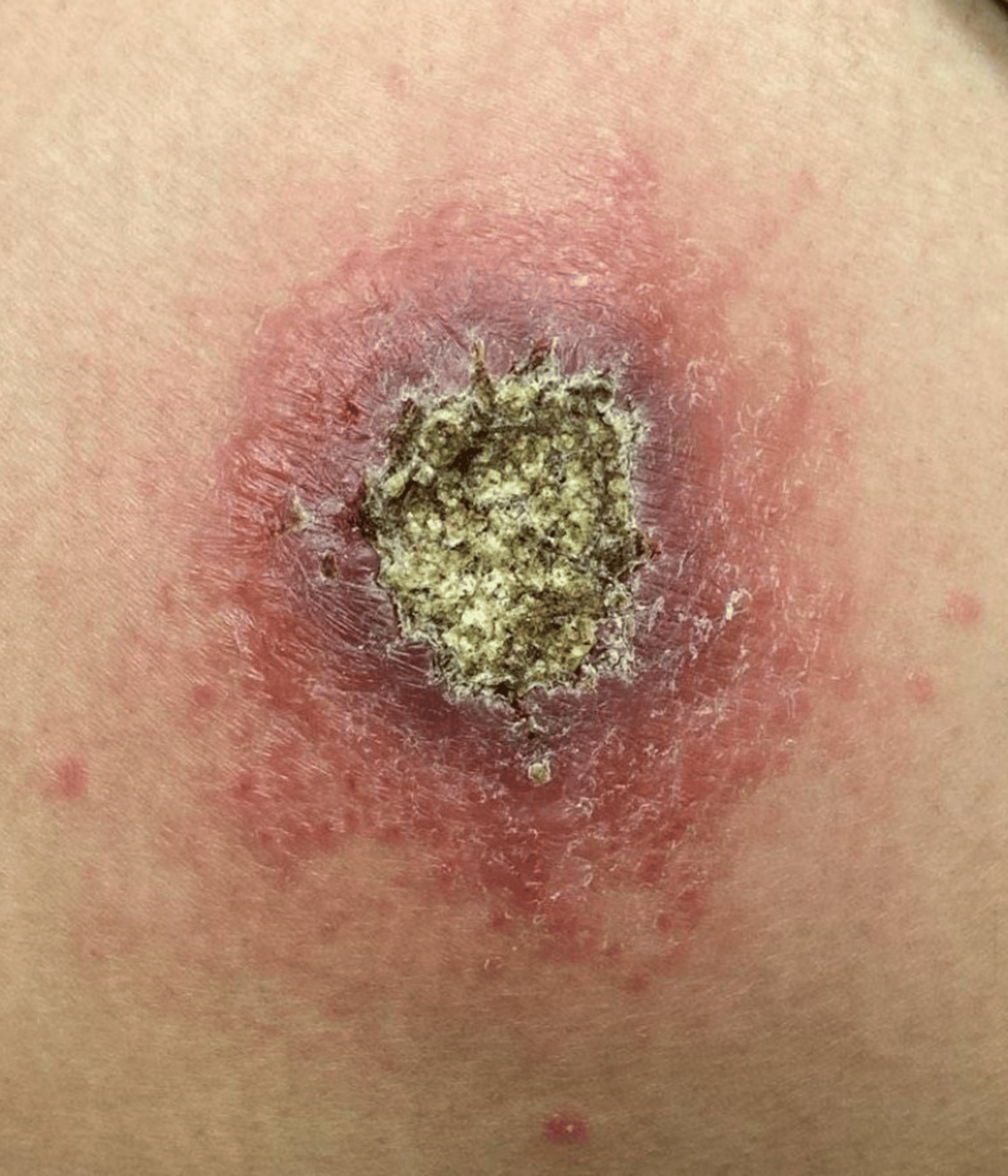
Ulcerated cutaneous leishmaniasis Ulcerated plaque on the left thigh with an erythematous border, central crusting, and satellite pink papules.

**Figure 2:**
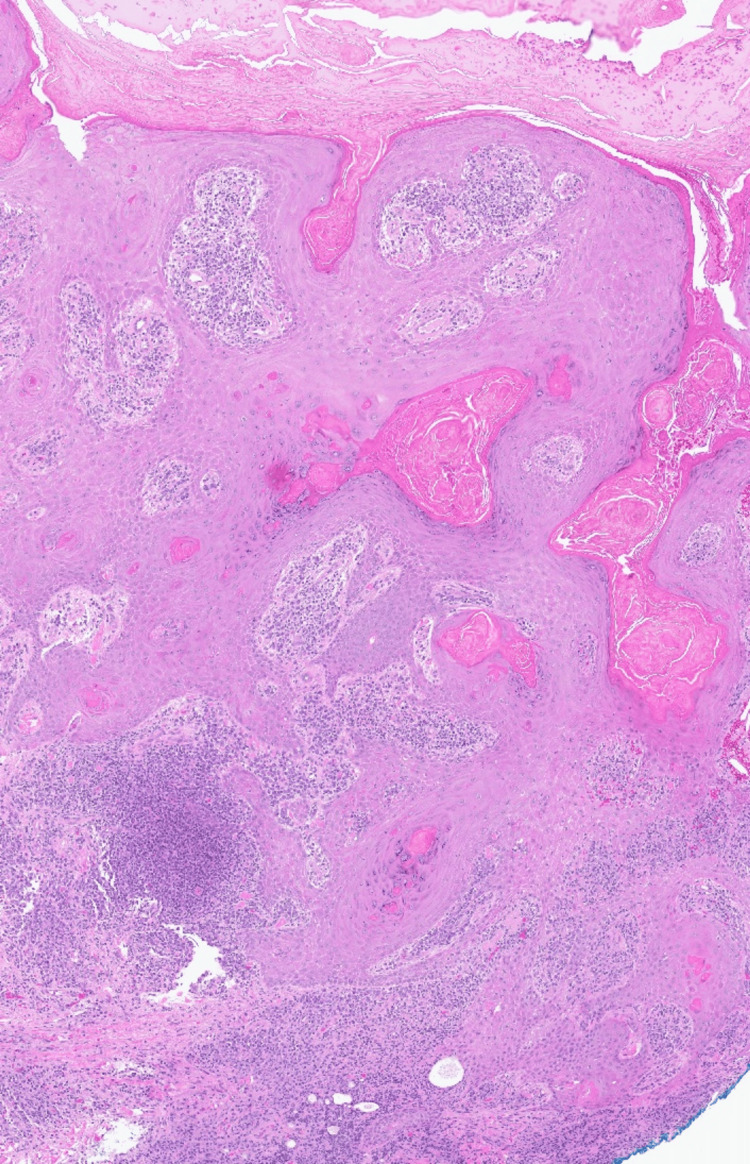
Histopathology findings Pathologic examination of a skin punch biopsy from the ulcer edge showing pseudoepitheliomatous hyperplasia, overlying crust, and dense dermal mixed inflammation (H&E, 2× magnification).

**Figure 3:**
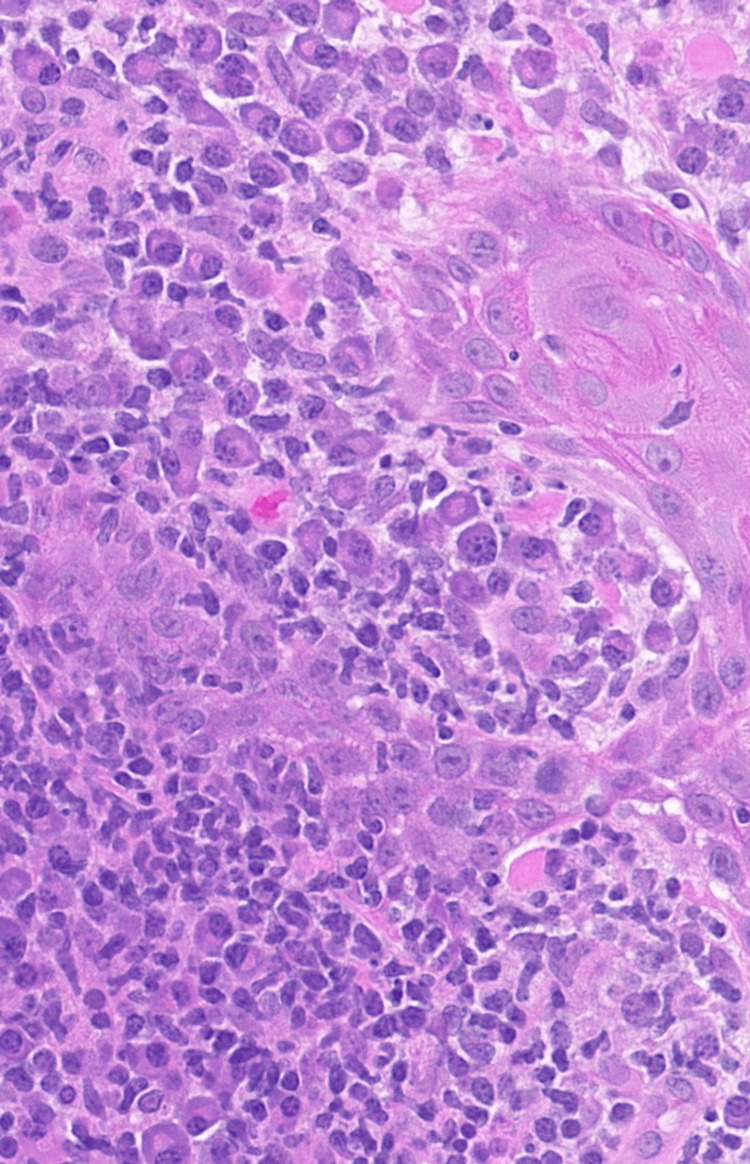
Lesional inflammatory infiltrate Higher magnification view of the lesional histopathology showing mixed dermal inflammation with a rich plasma cell component (H&E, 40× magnification).

Physical examination revealed a 6-cm ulcerated plaque with central crusting, peripheral erythema, and satellite pink papules on the left lateral thigh (Figure 1). Two punch biopsies of the ulcer edge and rim were taken for histopathologic examination and tissue culture. A tissue smear was negative for bacteria, fungi, and acid-fast bacilli. Histopathology revealed mixed dermal lymphoplasmacytic inflammation and reactive epidermal hyperplasia (Figure 2 and Figure 3). Various tissue stains (Fite, Giemsa, CD1a, acid-fast, Brown-Brenn, Gomori-methenamine-silver, and periodic acid-Schiff) failed to identify microbial pathogens in multiple specimen levels, and tissue cultures for bacteria, fungi, and mycobacteria were negative.

**Figure 4:**
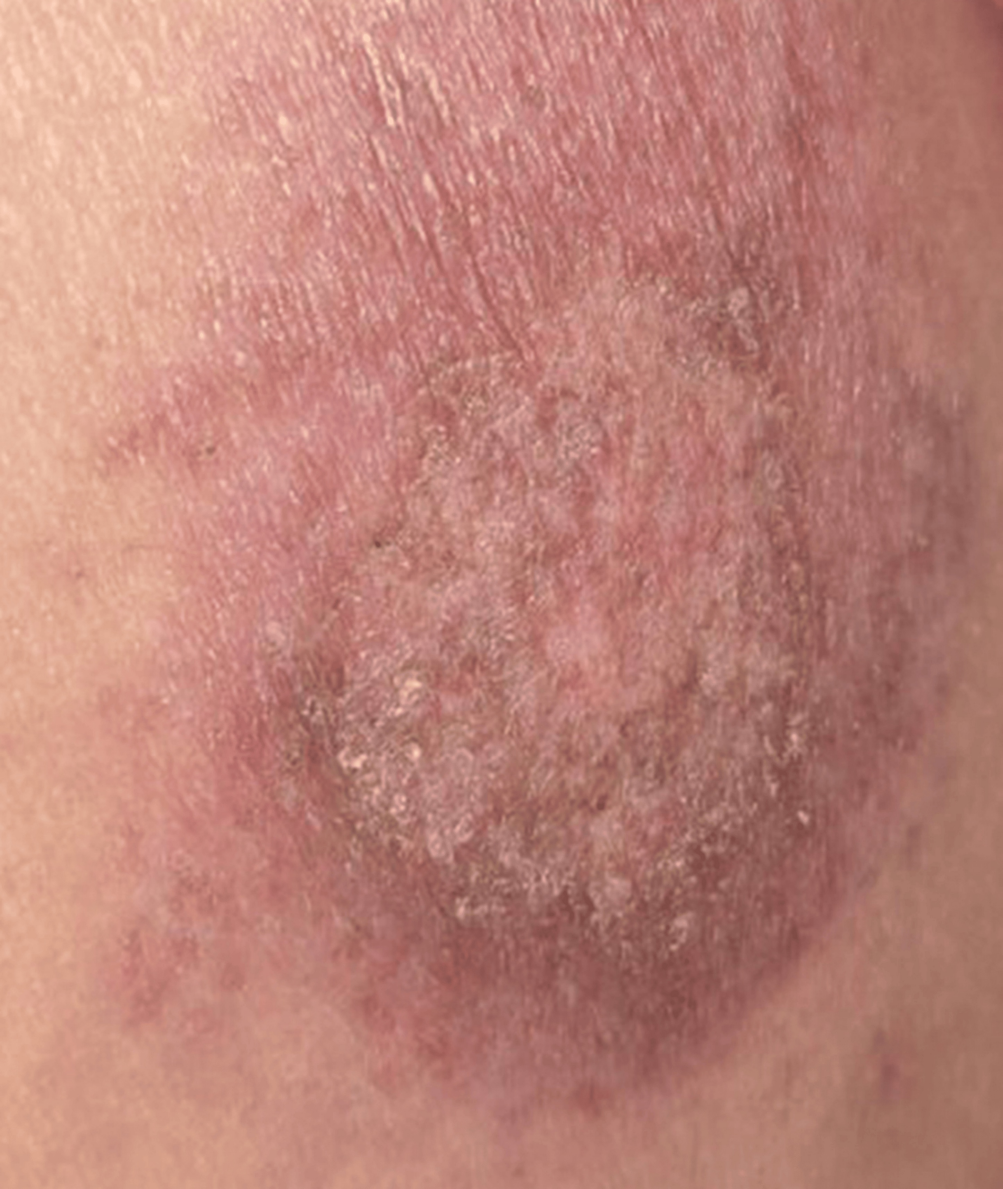
Post-treatment clinical image Two months after treatment with amphotericin B, the ulcer had completely healed, leaving dyspigmentation and scarring.

Due to high clinical suspicion based on the patient’s recent emigration from an endemic area for leishmaniasis, polymerase chain reaction (PCR) testing was performed on formalin-fixed skin biopsy sections, confirming the presence of *L. braziliensis*. After consultation with the Infectious Disease service, a peripherally inserted central catheter (PICC) was placed, and the patient was treated with intravenous liposomal amphotericin B 3 mg/kg daily for six days, consistent with CDC recommendations [11]. This treatment resulted in complete healing of the ulcer within two months (Figure 4).

## Discussion

Diagnosis of cutaneous leishmaniasis can be challenging in patients presenting in non-endemic regions, where prevalence and clinical suspicion are low. Cutaneous ulceration can also arise from a variety of pathogens, as exemplified by a published case in which cutaneous leishmaniasis was suspected due to patient travel history and sandfly exposure, but an ulcerative dermatophyte infection was ultimately diagnosed [12]. The differential diagnosis beyond leishmaniasis for chronic ulcerated cutaneous lesions is broad and includes ecthyma, deep fungal infections, cutaneous tuberculosis or atypical mycobacterial infection, pyoderma gangrenosum, or malignancy [5].

Although cutaneous *L. braziliensis* infection may be self-limiting in immunocompetent patients, treatment is recommended to limit ulcer size, extent of scarring, and risk of progression to mucosal disease [3,5,13]. Histopathologic examination of lesional biopsies can reveal intracellular pathogens, typically using Giemsa stain to highlight amastigote forms within macrophages. However, detecting pathogens in tissue sections can be challenging in immunocompetent patients, in whom parasites may be sparse or destroyed by immune cells [5,7]. The biopsy site may also affect diagnostic yield since parasite distribution in lesions is not uniform, though the border of the ulcer (including the inner edge) is the recommended site [14]. PCR-based testing for protozoal DNA is a highly sensitive diagnostic method, even with low parasite burden, and it also identifies the species to guide treatment [6,7,15,16]. When clinical suspicion is high based on potential exposure history and typical lesion morphology, Leishmania PCR testing can be pursued simultaneously with histopathology and tissue culture.

Treatment choice for cutaneous leishmaniasis is guided by lesion extent, propensity for dissemination, and the specific pathogen, as response to treatment varies significantly among species. First-line pentavalent antimonials have significant limitations, including a narrow therapeutic window, increasing resistance, limited availability, risk of relapse, and prolonged treatment duration [6,8,17]. Miltefosine is an effective FDA-approved first-line oral option for cutaneous leishmaniasis, but adverse effects such as gastrointestinal intolerance and high cost can limit its utility. Off-label intravenous liposomal amphotericin B is effective for treating cutaneous leishmaniasis caused by *L. braziliensis*, as in our case; however, this therapy requires PICC placement, multiple clinic visits for administration, and carries a risk of adverse effects, notably renal toxicity [13]. For some species, the widely available oral antifungal fluconazole can be effective, but its cure rate for *L. braziliensis* is low [6,18]. Treatment choice should be individualized, considering drug availability and cost, patient comorbidities, and regional species resistance.

## Conclusions

Cutaneous leishmaniasis remains a diagnostic and therapeutic challenge, especially in non-endemic regions where clinical suspicion is low. Increased human travel or displacement and climate-related environmental changes leading to vector spread have raised the likelihood of leishmaniasis presenting outside endemic regions. This case highlights the value of PCR for achieving a definitive diagnosis when conventional methods, such as tissue smear, histopathology stains, and cultures, are inconclusive. Early recognition and appropriate treatment are essential to prevent complications, including disfigurement from scarring or disease dissemination. Treatment is further complicated by potential toxicities, cost, and limited availability of therapeutic options, as well as rising pathogen resistance, emphasizing the need for research into this neglected tropical disease to identify therapies that are safer, cost-effective, and widely accessible.
